# Recursive Partitioning Analysis of Mediastinal N2 Lymph Node Involvement with Selected Biological Markers in Operable Non-small Cell Lung Cancer: A Correlative Study

**Published:** 2007-10-06

**Authors:** H Bozcuk, A Gumus, G Ozbilim, A Sarper, I Kucukosmanoglu, M Ozdogan, A Demircan

**Affiliations:** 1 Hakan Bozcuk, Mustafa Ozdogan; Akdeniz University Medical Faculty, Deparment of Medical Oncology, Antalya, Turkey; 2 Aziz Gumus; Akdeniz University Medical Faculty, Deparment of Internal Medicine, Antalya, Turkey; 3 Gulay Ozbilim, Kucukosmanoglu Ilknur; Akdeniz University Medical Faculty, Deparment of Pathology, Antalya, Turkey; 4 Alpay Sarper, Abit Demircan; Akdeniz University Medical Faculty, Deparment of Thoracic Surgery, Antalya, Turkey

**Keywords:** non-small cell lung cancer, P-glycoprotein, P53, c-erb-B2, recursive partitioning analysis, regression analysis, surgery

## Abstract

**Background:**

Expressions of various biomarkers in non-small cell lung cancer (NSCLC) have been linked with the prognosis and involvement of mediastinal lymph nodes.

**Methods:**

In this study, we utilized recursive partitioning analysis (RPA) by using P53, c-erb-B2, and P-glycoprotein (PGP) expressions evaluated by immunohistochemistry to estimate retrospectively the likelihood of the occult N2 mediastinal lymph node involvement in patients with operable NSCLC.

**Results:**

In univariate tests, immunohistochemical staining of the primary tumor for these 3 markers in 61 patients undergoing surgery revealed no direct relationship with the N2 involvement. However, RPA demonstrated in patients aged <75 and with ≥4 mediastinal lymph nodes removed that, high PGP expression frequency (≥20%) predicted an increased likelihood of the N2 involvement (46.7%, R^2^ = 0.25). Univariate nominal logistic regression analysis revealed that RPA group affiliation, and the number of mediastinal lymph nodes resected (logarithmic transformation) were associated with the metastasis to N2 lymph nodes (χ^2^ = 17.59, p = 0.0005, and χ^2^ = 2.40, p = 0.0654, respectively). Multivariate analysis confirmed that only RPA group affiliation predicted the N2 involvement (χ^2^ = 14.63, p = 0.0022).

**Conclusion:**

This study shows for the first time that PGP expression of the primary tumor may help to predict the occult N2 mediastinal lymph node involvement in NSCLC. Thus, further research is required to understand whether PGP expression may aid in the decision process for preoperative mediastinoscopy.

## Background

NSCLC is a biologically diverse disease with over a hundred prognosticators unveiled so far [1]. Surgery is still the mainstay of treatment for the early stages of NSCLC, but, survival is still poor with surgery only for locally advanced disease: 5 year overall survival is 10%–15% in clinical microscopic N2, and 2%–5% in clinical macroscopic N2 diseases [2]. Factors like serum-soluble intercellular adhesion molecule-1(ICAM-1) [3] and CEA levels, T stage, but not the mediastinal lymph node diameter on CT scan [4], expression of Caveolin-1, a tumor suppressor [5], and amplification of c-myc gene [6] have all been found to correlate with the presence of N2 disease, some of them also implying worse prognosis after surgery. In a previous study, our group showed in operable NSCLC that, different relationships of P53 (a tumor suppressor), c-erb-B2 (an oncogene) and PGP (P-glycoprotein: a product of the multidrug resistance gene; MDR1), with the patient outcome may exist for different tumor histologies, again highlighting the heterogeneity in biological behavior [7]. In this study, we wanted to explore whether these 3 biomarkers also helped to predict the likelihood of occult N2 disease, in order to find a new indication for mediastinoscopy.

## Methods

### Selection of patients

The patients were retrospectively selected from the database of the Thoracic Surgery department of Akdeniz University Medical Faculty Hospital, and all had curative resection for NSCLC. These patients, according to the guidelines of the treating institution, did not have clinical N2 or N3 disease by preoperative CT scans, and were deemed to have resectable disease by the treating surgeon. Patients had to have either mediastinal lymph node dissection or sampling at the time of surgery for inclusion into this study. All patients were staged with the help of chest and upper abdominal CT scanning, and in stage 3 disease bone scintigraphy was also requested. Only the patients operated within the thoracic surgery department at the Akdeniz University Hospital during the last 5 years (from 1.1.1998 to 31.12.2002) and with enough paraffin blocks remaining to perform all of the immunohistochemical studies in this study were retrospectively evaluated. Presence of nodal metastasis was evaluated by hematoxylin and eosin staining. The patients included in this study were from a subgroup of the previously published cohort from our center [7].

### Immunohistochemistry

Five μm sections of paraffin blocks containing tumor tissue were deparaffinised and dehydrated, and then subjected to the procedure of antigen retrieval. Immunohistochemical staining for PGP, P53 and c-erb-B2 was conducted by using Dako (1/100 dilution), Neomarker (1/50 dilution), and Neomarker (1/300 dilution), respectively. Expression of the biomarkers were defined both by *expression intensity* (“0 to +”; none or minimal, and “++ to +++”; moderate or strong) if at least 5% of the cells were stained, or by *expression frequency*, defined as the ratio of positively staining cells among a total of tumor cells.

### Statistical analysis

The immunohistochemical associates of N2 lymph node involvement were assessed by non-parametric tests, and by univariate logistic regression analysis. N2 lymph node involvement was chosen as the categorical response variable, and patient, disease factors and biomarker factors as predictor variables in the recursive partitioning analysis (RPA). In RPA, G^2 indicated the likelihood-ratio chi-square, which is actually twice the [natural log] entropy. Entropy is ∑-log (p) for each observation, where p is the probability (Prob) attributed to the response that occurred. The recursive partitioning analysis group membership, as well as the other variables in question were then also evaluated by univariate and multivariate nominal logistic regression analysis. Variables with a p value of <0.10 in univariate logistic regression analysis were subjected to multivariate logistic regression analysis. Receiver operating curve (ROC) analysis was employed to show the accuracy of the classification system based on RPA group affiliation to predict N2 involvement. A p value of <0.05 was accepted to be significant. JMP 5.0.1a (a business unit of SAS) was employed for the recursive partitioning analysis, where as, SPSS 11.5.0 (SPSS Inc., Chicago, IL, USA) was used for the logistic regression analysis.

## Results

### General features

A total of 61 cases with a median age of 65, and 86.9% male were selected for this study. A median of 8.6 lymph nodes were resected per each surgery. Occult N2 lymph node involvement was seen in 16 cases (26.3%). PGP, c-erb-B2, and P53 were expressed in 42.7%, 49.3%, and 48.8% of tumor cells, respectively. T1N0, T2N0 and T3N0 stages were encountered in 5 (8%), 20 (33%) and 8 (13%) cases. Please, see Table 1 for details. The expression intensity and frequency of these 3 biomarkers were not associated with the mediastinal N2 lymph node involvement (details given in Table 2).

### Recursive partitioning analysis of mediastinal N2 lymph node involvement

RPA analysis formed 4 groups of clinical relevance after 3 splits: **1**; age <75, LN ≥4, PGP ≥20% (N2 probability = 46.7%), **2**; age <75, LN ≥4, PGP <20% (N2 probability = 15.4%), **3**; age <75, LN <4 (N2 probability = 0%), **4**; age ≥75 (N2 probability = 0%). Thus, in age <75, LN ≥4 patients, PGP expression ≥20% predicted a 32% more likelihood of an associated N2 disease as opposed to those with PGP expression <20%. This model had an R^2^ of 0.25.

**Figure 1:**
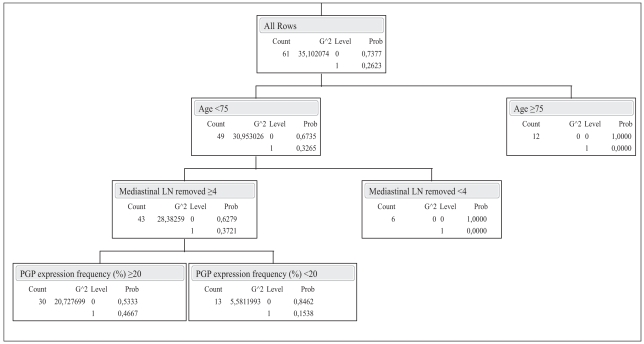
Tree view and leaf report for the recursive partitioning analysis model of mediastinal N2 lymph node involvement. LN: Lymph node, G^2: the likelihood-ratio chi-square. Prob: the probability attributed to the response that occurred.

**Figure 2:**
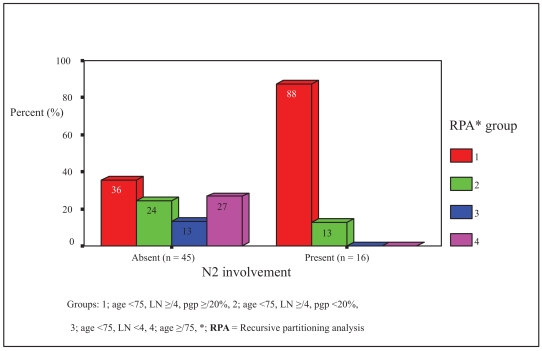
Recursive partitioning analysis group and mediastinal N2 lymph node involvement.

Likewise, in patients with N2 disease, RPA group 1 (age <75, LN ≥4, pgp ≥20%) comprised 88% of cases, as opposed to 36% in those without N2 disease. (Refer to Figures 1 and 2 for details of RPA).

The corresponding sensitivity, specifity, false negative rate, and false positive rate of the model were 47%, 89%, 14%, and 46%, respectively. In addition, the associated positive and negative predictive values for this model were 54% and 86%, in order.

Receiver operating curve (ROC) analysis yielded an area of 0.79 for N2 status prediction with RPA group affiliation.

### Logistic regression analysis for determinants of mediastinal N2 involvement

Univariate nominal logistic regression analysis showed that RPA group affiliation, and the number of mediastinal lymph nodes resected (logarithmic transformation) were linked with the involvement of N2 lymph nodes (χ^2^ = 17.59, p = 0.0005, and χ^2^ = 2.40, p = 0.0654, respectively). Multivariate analysis also showed that RPA group affiliation was the only predictor of N2 involvement (χ^2^ = 14.63, p = 0.0022). The details of logistic regression analysis are displayed in Table 2.

## Discussion

We show in this study by RPA that the PGP expression was linked with the mediastinal N2 lymph node involvement in a subgroup (43/61; 71%) of patients with operable NSCLC. Studies in the literature have yielded conflicting results on the prognostic role of PGP in NSCLC. The 1st one claimed of no prognostic role, whereas the 2nd one showed better prognosis with the increased expression of PGP [8, 9]. A previous report from our group was the 3rd one, and to our knowledge also the last one in the literature, that similarly showed no prognostic role for PGP in NSCLC [7]. To the best of our knowledge, there is no information in NSCLC on the relationship of PGP expression with the mediastinal lymph node involvement. This study shows a higher likelihood of N2 disease with higher expression of PGP. Interestingly, this relationship is not evident in the univariate analyses, but in the RPA. It is possible to speculate that high PGP expression in NSCLC may imply a more aggressive clinical behavior of cancer cells. This aggressive clinical behavior is possibly mediated via different molecules such as Y-box binding protein-1, as was also shown in breast cancer [10].

We also demonstrated that younger age and higher number of mediastinal lymph nodes dissected were linked with the presence of N2 disease. This is in line with the fact that younger age is not a good prognosticator in NSCLC patients treated with surgery [11, 12]. Removing more than 3 mediastinal lymph nodes also apparently make it easier and more probable to catch microscopically involved N2 lymph nodes. In accordance, Keller et al also showed that with systematic mediastinal lymph node dissection, it was more probable to detect multilevel N2 disease [13].

There may be a number of shortcomings with this study; namely its retrospective nature and small size. However, we think the hypothetic role proposed in this study for PGP to predict N2 disease warrants further research. In addition, although the proposed recursive partitioning model had good negative predictive value (86%), its sensitivity should still be considered suboptimal (47%). Thus, this model may theoretically suggest those patients who may be spared from mediastinoscopy, but it still lacks power to identify the subgroup of patients who may need mediastinoscopy.

In short, our findings suggest that even when clinical N2 disease is absent, PGP expression of the primary tumor may alert clinicians towards preoperative mediastinoscopy. Although, the general recommendation for microscopic N2 disease discovered at the time of surgery is surgical resection [14], we do not know whether neoadjuvant chemotherapy improves survival in this specific group of patients. Further larger clinical trials of prospective nature are needed to understand the importance of PGP expression to guide preoperative mediastinoscopy, and to select the optimal treatment for microscopic N2 disease.

## Conclusion

PGP expression of the primary tumor in operable NSCLC may prove useful in the prediction of N2 disease. In this regard, prospective and larger clinical studies are needed to conclude further.

## Figures and Tables

**Table 1:** Patient, disease and immunohistochemical features.

Features	n(%)	Median/Mean	Range
Total	61 (100.0)		
*Patient factors*			
Age		65.0/65.2	44–84
Gender			
Female	8 (13.1)		
Male	53 (86.9)		
Amount of smoking (pack-years)		45.0/46.9	0–100
*Disease factors*			
Histology			
Squamous cell carcinoma	39 (63.9)		
Non-squamous cell carcinoma	22 (36.1)		
T status			
1	8 (13.1)		
2	43 (70.5)		
3	9 (14.8)		
4	1 (1.6)		
N status			
N0 and N1	45 (73.7)		
N2	16 (26.3)		
Number of mediastinal lymph nodes resected		7.0/8.6	1–39
*Immunohistochemical factors*			
PGP expression intensity*		1/1.4	0–3
PGP expression frequency (%)**		40/42.7	0–95
c-erb-B2 expression intensity*		1/1.4	0–2
c-erb-B2 expression frequency (%)**		60/49.3	0–90
P53 expression intensity*		2/1.7	0–3
P53 expression frequency (%)**		50/48.8	0–95

* magnitude of staining (0 to +++, over a scale of 3)

** % tumor cells stained with the specific marker

**Table 2:** Determinants of N2 mediastinal lymph node involvement.

Parameter	Univariate tests		Multivariate tests	
		
	LR_χ^2^_*	p	LR_χ^2^_*	p
*Patient factors*				
Age	0.32	0.5711		
Gender (Female vs male)	0.01	0.9321		
Amount of smoking (pack-years)	0.13	0.7233		
*Disease factors*				
Histology (Squamous vs non-squamous cell carcinoma)	0.02	0.8896		
T status (ordinal variable from 1 to 4)	5.19	0.1581		
Number of mediastinal lymph nodes resected (logarithmic transformation)	2.40	0.0654	0.44	0.5077
*Immunohistochemical factors*				
PGP expression intensity**	0.09	0.7699		
PGP expression frequency (%)***	0.74	0.3913		
c-erb-B2 expression intensity**	0.12	0.7348		
c-erb-B2 expression frequency (%)***	0.09	0.7693		
P53 expression intensity**	0.32	0.5708		
P53 expression frequency (%)***	0.01	0.9276		
*Recursive partitioning analysis group*$	17.59	0.0005	14.63	0.0022

* Likelihood ratio Chi square

** magnitude of staining (0 to +++, over a scale of 3)

*** % tumor cells stained with the specific marker

$ Recursive partitioning analysis group affiliation (of the 4 leaves formed by the model)
